# Development and validation of a clinical nomogram for differentiating hemorrhagic and ischemic stroke prehospital

**DOI:** 10.1186/s12883-023-03138-1

**Published:** 2023-03-03

**Authors:** Sheng Ye, Huiqing Pan, Weijia Li, Jinqiang Wang, Hailong Zhang

**Affiliations:** 1 Department of Emergency Medicine, The Second Affiliated Hospital of Wannan Medical College, Wuhu, Anhui China; 2 Emergency Sub-Station, The Second Affiliated Hospital of Wannan Medical College, Wuhu, Anhui China; 3 School of Clinical Medicine, Wannan Medical College, Wuhu, Anhui China; 4 Department of Emergency Medicine, Wuhu Emergency Medical Center, Wuhu, Anhui China

**Keywords:** Hemorrhagic stroke, Ischemic stroke, Nomogram, Emergency medical services, Prehospital

## Abstract

**Objectives:**

The early detection and identification of stroke are essential to the prognosis of patients with suspected stroke symptoms out-of-hospital. We aimed to develop a risk prediction model based on the FAST score to identify the different types of strokes early for emergency medical services (EMS).

**Methods:**

This retrospective observational study enrolled 394 stroke patients at a single center from January 2020 to December 2021. Demographic data, clinical characteristics, and stroke risk factors with patients were collected from the EMS record database. Univariate and multivariate logistic regression analysis was used to identify the independent risk predictors. The nomogram was developed based on the independent predictors, in which the discriminative value and calibration of the nomogram were verified by the receiver operator characteristic (ROC) curve and calibration plots.

**Results:**

A total of 31.90% (88/276) of patients were diagnosed with hemorrhagic stroke in the training set, while 36.40% (43/118) in the validation set. The nomogram was developed based on the multivariate analysis, including age, systolic blood pressure, hypertension, vomiting, arm weakness, and slurred speech. The area under the curve (AUC) of the ROC with nomogram was 0.796 (95% CI: 0.740–0.852,* P* < 0.001) and 0.808 (95% CI:0.728–0.887, *P* < 0.001) in the training set and validation set, respectively. In addition, the AUC with the nomogram was superior to the FAST score in both two sets. The calibration curve showed a good agreement with the nomogram and the decision curves analysis also demonstrated that the nomogram had a wider range of threshold probabilities than the FAST score in the prediction risk of hemorrhagic stroke.

**Conclusions:**

This novel noninvasive clinical nomogram shows a good performance in differentiating hemorrhagic and ischemic stroke for EMS staff prehospital. Moreover, all of the variables of nomogram are acquired in clinical practice easily and inexpensively out-of-hospital.

## Background

Stroke is a severe manifestation of cardiovascular disease, which leads to the second cause of death in the world [1]. Nearly 20 million people experience stroke annually, and the incidence is increasing because of the aging of the population [1, 2]. The ischemic stroke makes up about 88% of all strokes, and the remainder is hemorrhagic stroke [3, 4]. At present, endovascular therapy and intravenous tissue-type plasminogen activators are the most effective therapies for acute ischemic stroke in the therapeutic time window [5]. In addition, receiving operation and intervention treatment in time for hemorrhagic stroke is crucial [6]. Thus, it is critical to improve the prognosis of stroke patients for establish the stroke center network and stroke “Green Channel”, which shortens the treatment time and integrates the medical resources.

Almost 50% of stroke occurred out-of-hospital. Emergency medical services (EMS) are the first point to contract patients who appear suspected stroke symptoms [7]. Early identification of ischemic or hemorrhagic stroke from stroke patients could provide earlier diagnosis, referral to the appropriate emergency department, and given a better treatment decision [8]. Therefore, distinguishing hemorrhagic stroke from ischemia stroke has an important implication for EMS.

Some prehospital stroke scales were used for EMS to identify stroke in recent studies, such as the Face Arm Speech Test (FAST), Los Angeles Prehospital Stroke Screen (LAPSS), and Recognition of Stroke in the Emergency Room (ROSIER), parts of which had moderate-to-good sensitivity and the lower levels of specificity [9–12]. In addition, some studies have proved that serum markers had a certain advantage in distinguishing hemorrhagic stroke and ischemic stroke, including glial fibrillary acidic protein (GFAP), N-terminal proB-type natriuretic peptide (NT-proBNP), and retinol-binding protein 4 (RBP-4) [13, 14]. However, the difficulty to detect prehospital limited their application.

Therefore, this study aims to establish a simple and reliable clinical tool to identify ischemic and hemorrhagic stroke prehospital based on the easy-to-obtain prehospital clinical data and FAST scale. Moreover, the use of the tool would be convenient for EMS staff to identify stroke types early and improve emergency efficiency in future clinical applications.

## Methods

### Study design and participants

This retrospective observational study enrolled patients with suspected stroke symptoms onset from January 1, 2020 to December 1, 2021 in the second affiliated hospital of Wannan Medical College. All patients were admitted to the emergency department by the EMS. The study was approved by the Ethics Committee of the Second Affiliated Hospital of Wannan Medical College (Number: wyefyls202205) and followed the principles of the Declaration of Helsinki. The informed consent of patients was waived due to the retrospective observational design.

Participants of the following criteria were included in this study: 1) age > 18 years; 2) admission to the emergency department with a suspected diagnosis of stroke; 3) computed tomography (CT) or magnetic resonance imaging (MRI) scan during hospitalization. The exclusion criteria were:1) patients using EMS as a secondary transport; 2) data missed with EMS records; 3) patients died before CT or MRI was performed; 4) stroke caused by trauma.

**Fig. 1:**
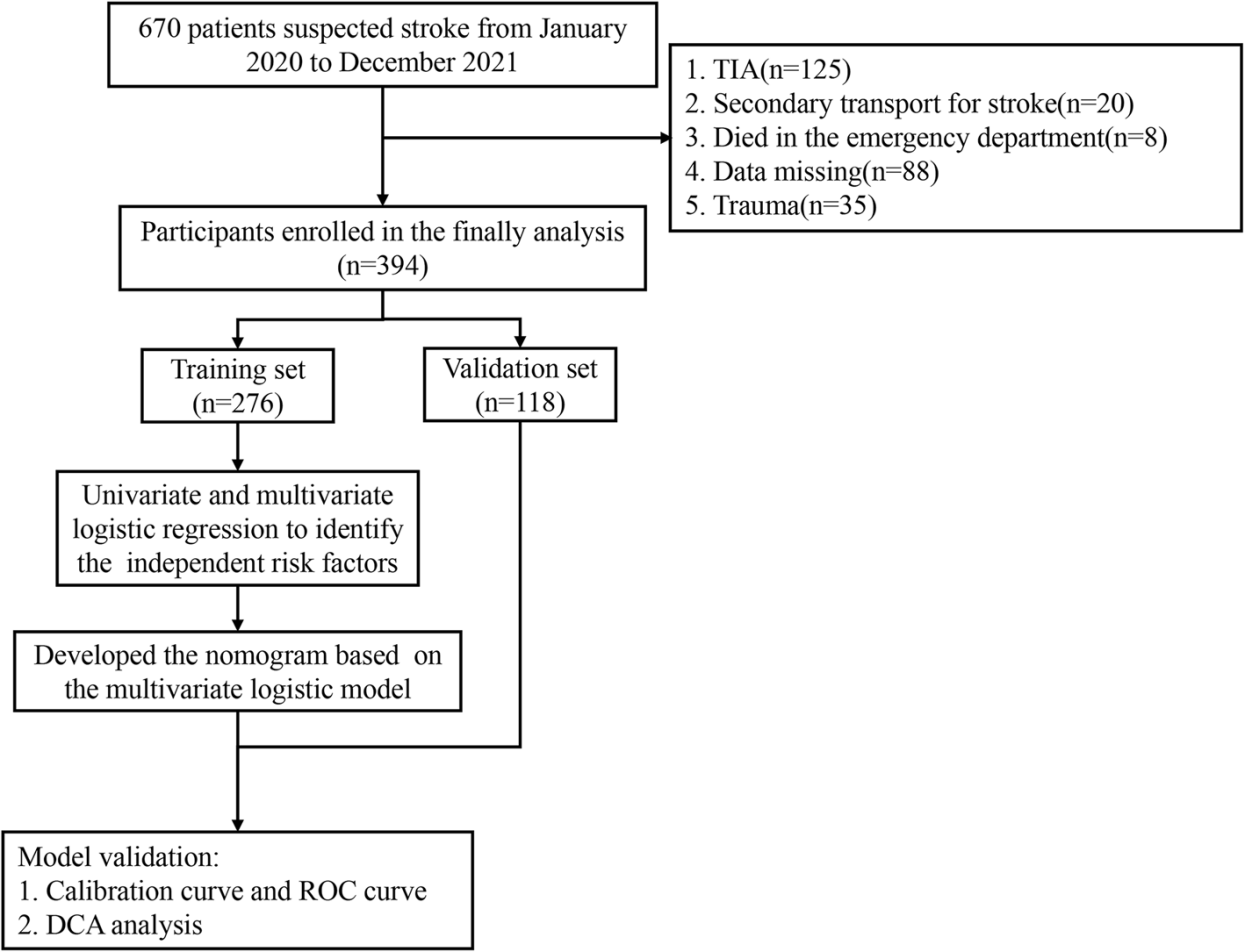
Flow chart with study

A total of 670 patients were included in this study. Finally, 394 patients were enrolled in the final analysis according to the inclusion and exclusion criteria. All patients were divided into a training set (*n* = 276) and a validation set (*n* = 118) in a ratio of 7:3 (Fig. 1).

### Data collection

Baseline demographic data, prehospital clinical characteristics, and stroke risk factors were collected from the EMS record database in the Wuhu Emergency Medical Center, including age, gender, blood pressure, clinical features, diabetes, hypertension, duration of symptom, etc. The prehospital vital signs (blood pressure and heart rate) were measured using an electrocardiogram blood pressure monitor (Mindray, China) by EMS personnel out-of-hospital.

### Definition

Hemorrhagic stroke was defined as a new symptomatic neurologic deterioration accompanied by neuroimaging evidence of extravasation of blood into the brain parenchyma spontaneous and non-traumatic, including intraparenchymal hemorrhage and subarachnoid hemorrhage [15, 16]. Ischemic stroke was defined as a sudden neurologic dysfunction with imaging evidence of acute infarction by MRI or CT scan [17]. Transient cerebral ischemic attack (TIA) was defined as new neurologic symptoms lasting less than 24 h with no new infarction or hemorrhage on neuroimaging [18]. The Face Arm Speech Test (FAST) contained items that unilateral facial droop (F) or arm weakness (A) and Slurred speech (S) [19]. Duration of symptom was defined as the duration since suspected stroke symptoms onset until the EMS arrived.

### Statistical analysis

All statistical analyses were performed using R software (version 3.6.2, http://www.r-project.org) and SPSS software (version 22.0, IBM, New York, USA). Kolmogorov–Smirnov(K-S) test was used to discriminate normal distribution for all continuous variables. Continuous variables were expressed as mean ± standard deviation (SD) or interquartile range (IQR), which depended on the normal distribution. Categorical variables were expressed as numbers (percentages). Differences between the two groups for continuous variables were analyzed by the student’s test or Mann–Whitney U test, and the chi-squared test was used for categorical variables. Multivariate logistic regression models with the entering method were accomplished to determine the independent risk predictors of hemorrhagic stroke in the nomogram. The variables included factors with significance at *P* < 0.1 in the univariate analysis. Moreover, the final total score of the nomogram was constructed by a preliminary score of each predictor with a point ranging from 0–100, which was converted to the probability of hemorrhagic stroke (0–100%). Calibration was implemented to assess the fitting degree between the actual and nomogram-predicted hemorrhagic stroke by using a calibration plot with bootstraps of 1000 resamples.

The area under the curve (AUC) of receiver operating characteristic (ROC) was used to compare the discrimination ability of nomogram and traditional FAST score. Decision curve analysis (DCA) was further performed to estimate the clinical value of the nomogram and traditional FAST score with the net benefit for multiple threshold probabilities. All accuracy estimates and regression coefficients reported 95% confidence intervals (CI) and a two-tailed *P* < 0.05 was considered statistically significant.

## Results

### Demographic and prehospital clinical characteristics of the study

A total of 394 patients who met the eligibility criteria were retrospectively enrolled during the period between Jan 2020 and Dec 2021. The incidence of hemorrhagic stroke in the training set was 31.90% (88/276), while 36.40% (43/118) in the validation set. There were no statistical differences between the two sets in all clinical characteristics (all *P* > 0.05) (Table 1).

**Table 1:** Clinical characteristics of patients in the training set and validation set

Variables	Total (*n* = 394)	Training set (*n* = 276)	Validation set (*n* = 118)	t/z/χ^2^	*P* value
**Demographic characteristics**					
Age, years (mean ± SD)	71.01 ± 14.54	70.69 ± 14.74	71.77 ± 14.09	-0.677	0.499
Male, n (%)	233(59.10)	163(59.10)	70(59.30)	0.002	0.961
BMI, kg/m^2^(mean ± SD)	21.06 ± 3.55	20.90 ± 3.49	21.44 ± 3.69	-1.375	0.170
**Prehospital clinical data**					
SBP, mmHg, IQR	132.00(119.75, 150.00)	134.00(118.00, 151.50)	130.00(120.00, 150.00)	-0.486	0.627
DBP, mmHg, IQR	80.00(68.00, 88.00)	80.00(70.00, 86.00)	79.50(64.75, 88.00)	-0.984	0.325
Heart rate, bpm, IQR	84.00(71.75, 100.00)	84.50(71.25, 100.00)	83.50(71.75, 100.00)	-0.231	0.817
**Past medical history**					
Hypertension, n (%)	108(27.40)	77(27.90)	31(26.30)	0.110	0.740
Diabetes mellitus, n (%)	80(20.30)	56(20.30)	24(20.30)	0.000	0.991
Atrial fibrillation, n (%)	86(21.80)	61(22.10)	25(21.20)	0.041	0.840
Coronary heart disease, n (%)	96(24.40)	69(25.00)	27(22.90)	0.201	0.654
Chronic renal failure, n (%)	73(18.50)	45(16.30)	28(23.70)	3.018	0.082
**Prehospital clinical symptoms**					
Headache, n (%)	140(35.50)	100(36.20)	40(33.90)	0.197	0.658
Dizzy, n (%)	186(47.20)	122(44.20)	64(54.20)	3.340	0.068
Vomiting, n (%)	147(37.30)	100(36.20)	47(39.80)	0.458	0.499
**FAST score**					
Facial droop, n (%)	170(43.10)	118(42.80)	52(44.10)	0.058	0.809
Arm weakness, n (%)	174(44.20)	122(44.20)	52(44.10)	0.001	0.980
Slurred speech, n (%)	155(39.30)	114(41.30)	41(34.70)	1.490	0.222
**Duration of symptom**, min, IQR	35.00(25.00, 50.00)	35.00(25.00, 50.00)	30.00(25.00, 51.25)	-0.322	0.747
**Stroke type**				0.773	0.379
Hemorrhagic stroke	131(33.20)	88(31.90)	43(36.40)		
Ischemic stroke	263(66.80)	188(68.10)	75(63.60)		

*BMI* Body mass index, *SBP* Systolic blood pressure, *DBP* Diastolic blood pressure, *Bpm* Beats per minute, *FAST* Face-Arm-Speech-Time, *IQR* Interquartile range

In the training set, the frequency of hypertension (*P* = 0.003) was higher in hemorrhagic stroke patients, compared to ischemic stroke patients. The incidence of headache (*P* = 0.029) and vomiting (*P* = 0.007) was significantly increased in hemorrhagic stroke. Meanwhile, a higher incidence of the FAST Scale (arm weakness, and slurred speech) was observed in hemorrhagic stroke patients (*P* = 0.004, and *P* = 0.002). In addition, patients with hemorrhagic stroke tended to have the lower age (*P* < 0.001) and higher systolic blood pressure (*P* = 0.006) (Table 2).

**Table 2:** Clinical characteristics of hemorrhagic and ischemic stroke patients in the training set

Variables	Total (*n* = 276)	Hemorrhagic stroke (*n* = 88)	Ischemic stroke (*n* = 188)	t/z/χ^2^	*P* value
**Demographic characteristics**					
Age, years (mean ± SD)	70.69 ± 14.74	63.70 ± 16.98	73.96 ± 12.31	-5.685	< 0.001
Male, n (%)	163(59.10)	52(59.10)	111(59.00)	0.000	0.994
BMI, kg/m^2^ (mean ± SD)	20.90 ± 3.49	21.25 ± 3.30	20.74 ± 3.57	1.133	0.258
**Prehospital clinical data**					
SBP, mmHg, IQR	134.00(118.00, 151.50)	143.50(120.00, 160.00)	130.00(116.25, 146.00)	-2.751	0.006
DBP, mmHg, IQR	80.00(70.00, 86.00)	80.00(70.00, 90.00)	80.00(68.00, 85.00)	-1.447	0.148
Heart rate, bpm, IQR	84.50(71.25, 100.00)	82.50(70.25, 102.75)	85.00(72.00, 98.75)	-0.083	0.934
**Past medical history**					
Hypertension, n ( %)	77(27.90)	35(39.80)	42(22.30)	9.056	0.003
Diabetes mellitus, n (%)	56(20.30)	14(15.90)	42(22.30)	1.533	0.216
Atrial fibrillation, n (%)	61(22.10)	18(20.50)	43(22.90)	0.204	0.652
Coronary heart disease, n (%)	69(25.00)	19(21.60)	50(26.60)	0.801	0.371
Chronic renal failure, n (%)	45(16.30)	14(15.90)	31(16.50)	0.015	0.903
**Prehospital clinical symptoms**					
Headache, n (%)	100(36.20)	40(45.50)	60(31.90)	4.756	0.029
Dizzy, n (%)	122(44.20)	42(47.70)	80(42.60)	0.651	0.420
Vomiting, n (%)	100(36.20)	42(47.70)	58(30.90)	7.389	0.007
**FAST score**					
Facial droop, n (%)	118(42.80)	44(50.00)	74(39.40)	2.772	0.096
Arm weakness, n (%)	122(44.20)	50(56.80)	72(38.30)	8.336	0.004
Slurred speech, n (%)	114(41.30)	48(54.50)	66(35.10)	9.343	0.002
**Duration of symptom**, min, IQR	35.00(25.00, 50.00)	30.00(25.00, 50.00)	35.00(25.00, 50.00)	-0.772	0.440

*BMI* Body mass index, *SBP* Systolic blood pressure, *DBP* Diastolic blood pressure, *Bpm* beats per minute, *FAST* Face-Arm-Speech-Time, *IQR* Interquartile range

### Multivariate logistic regression analyses of independent risk factors for hemorrhagic stroke

Multivariate logistic regression analysis was used to calculate the adjusted odds ratio (aOR) value of each independent risk factor in the training set (Table 3). After multivariable adjustment, systolic blood pressure (aOR:1.014, 95% CI: 1.002–1.025, *P* = 0.023), hypertension (aOR:2.440, 95% CI: 1.291–4.613, *P* = 0.006), age (aOR:0.942, 95% CI: 0.921–0.964, *P* < 0.001), and vomiting (aOR:2.741, 95% CI: 1.465–5.129, *P* = 0.002) remained significant after adjusting for confounders. Besides, arm weakness (aOR:2.559, 95% CI: 1.397–4.687, *P* = 0.002) and slurred speech (aOR:2.072, 95% CI: 1.142–3.760, *P* = 0.017) were independently associated with hemorrhagic stroke.

**Table 3:** Univariate analysis and multivariate logistic regression analysis for the risk factors associated with hemorrhagic stroke in the training set

Variables	Unadjusted OR (95% CI)	*P*-value	Adjusted OR (95% CI)	*P-*value
Age, years	0.951(0.932–0.970)	< 0.001	0.942(0.921–0.964)	< 0.001
SBP, mmHg	1.015(1.004–1.025)	0.005	1.014(1.002–1.025)	0.023
Hypertension	2.296(1.327–3.970)	0.003	2.440(1.291–4.613)	0.006
Headache	1.778(1.057–2.989)	0.030	1.408(0.761–2.607)	0.276
Vomiting	2.046(1.216–3.443)	0.007	2.741(1.465–5.129)	0.002
Facial droop	1.541(0.925–2.566)	0.097	1.756(0.959–3.218)	0.068
Arm weakness	2.120(1.268–3.545)	0.004	2.559(1.397–4.687)	0.002
Slurred speech	2.218(1.325–3.714)	0.002	2.072 (1.142–3.760)	0.017

*SBP* Systolic blood pressure, *OR* Odds ratio, *CI* Confidence intervals

### Establishment of nomogram in predicting hemorrhagic stroke

**Fig. 2:**
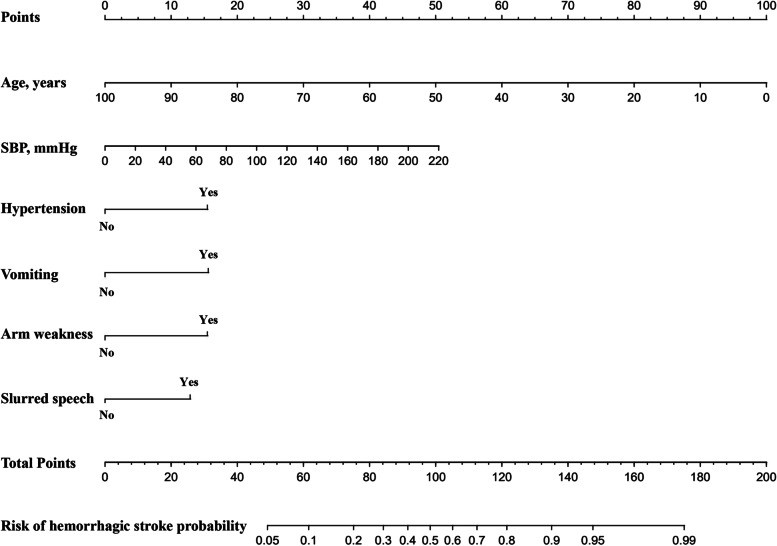
The nomogram for predicting hemorrhagic stroke probability based on the 6 independent risk factors

**Fig. 3:**
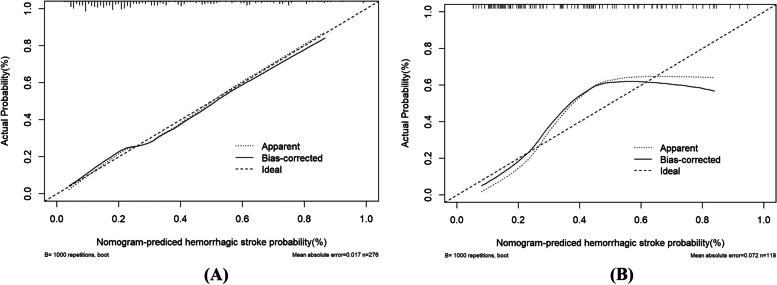
Calibration curves of the nomogram in the training set and the validation set. Notes: **A** the nomogram in the training set (*n* = 276); **B** the nomogram in the validation set (*n* = 118). The y-axis represents the observed rate of hemorrhagic stroke, and the x-axis represents the nomogram-predicted probability of hemorrhagic stroke. The dotted lines represented by the nomogram are closer to the diagonal grey lines representing a better prediction

A clinical nomogram to predict hemorrhagic stroke was developed based on multivariate logistic regression analysis using the 6 independent risk factors. Each of the major significant predictors was assigned with points ranging from 0 to 100, and the preliminary scores were summarized as the total predictive score. Based on the total score, a visualized percentage to predict the risk of hemorrhagic stroke was shown in the nomogram (Fig. 2). The calibration plots with 1000 Bootstrap resamples were described, which demonstrated a good-predictive performance between the predicted probability of hemorrhagic stroke and the actual observations, with data points on the plots close to the ideal curve (Fig. 3).

### Validation of nomogram with receiver operating characteristic (ROC) curve

The AUC of ROC was analyzed to investigate the discrimination of the nomogram, which was 0.796 (95% CI:0.740–0.852, *P* < 0.001) in the training set and 0.808 (95% CI:0.728–0.887, *P* < 0.001) in the validation set.

**Fig. 4:**
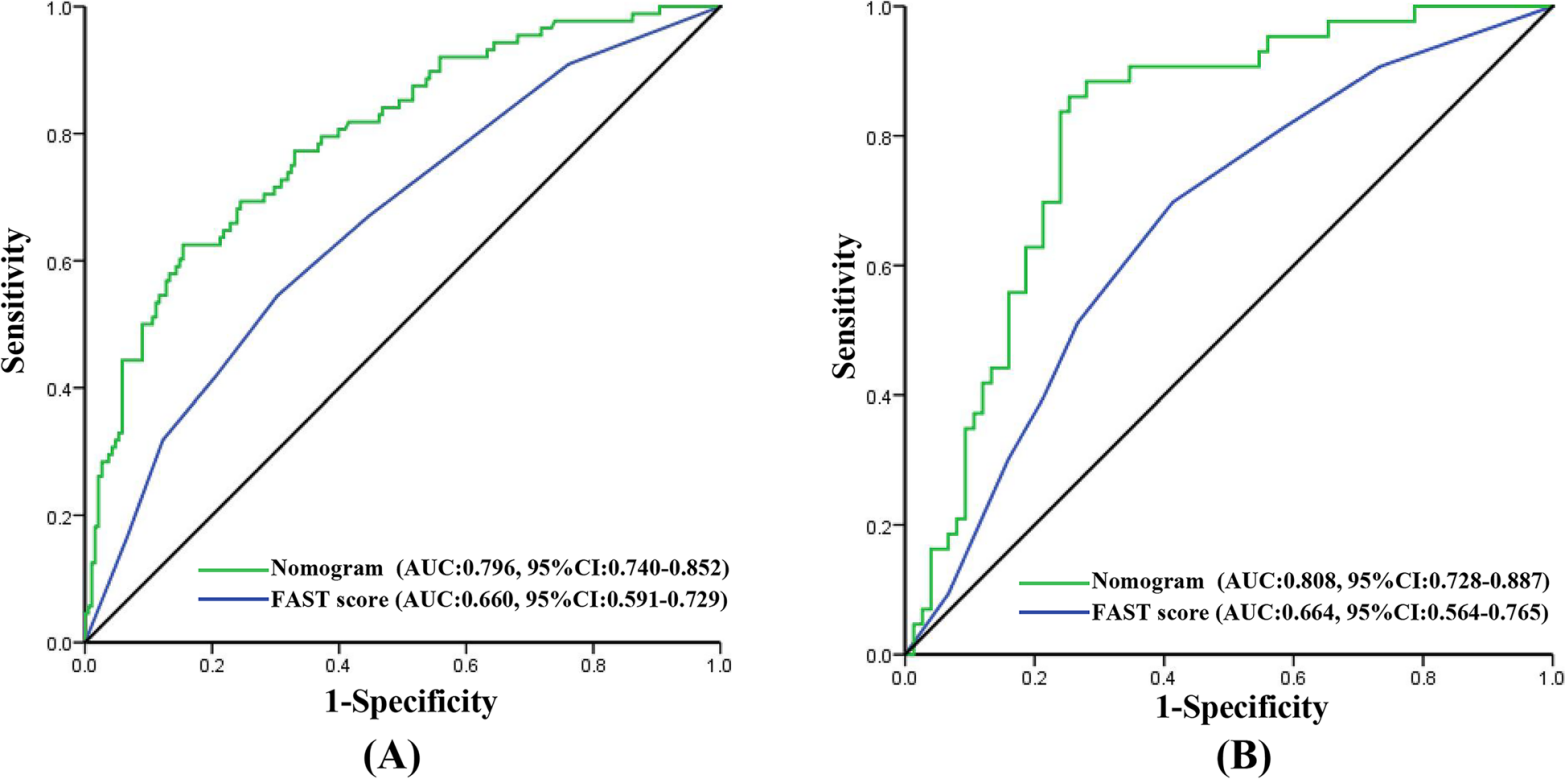
The ROC curve of the nomogram and the FAST score in the training set and the validation set. Notes: **A** ROC in the training set; **B** ROC in the validation set. The green line represents the ROC curve of the nomogram and the blue line represents the ROC curve of the FAST score

Furthermore, we compared the discrimination between the nomogram and the FAST score, and the results indicated the AUC of the nomogram was superior to that of the FAST score in both the training set (AUC = 0.796 vs 0.660, *P* < 0.001) and validation set (AUC = 0.808 vs 0.664,* P* = 0.004), indicating the novel nomogram had higher predictive efficiency (Fig. 4).

### Clinical use compared nomogram with the FAST score

**Fig. 5:**
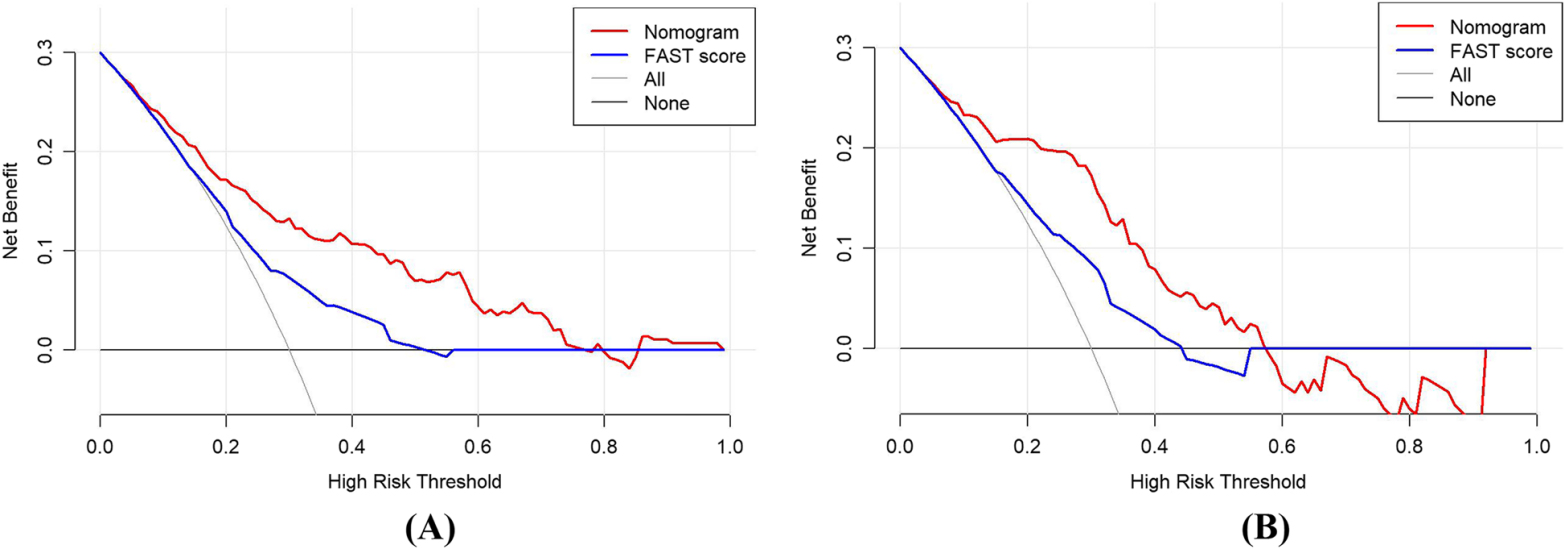
The decision curves analyses (DCA) of the nomogram and the FAST score in the training set and validation set. Notes: **A** DCA in the training set; **B** DCA in the validation set. The red line represents the DCA curve of the nomogram and the blue line represents the DCA curve of the FAST score

Decision curve analysis (DCA) curves were applied to compare the clinical validity of the nomogram and the FAST score, suggesting that the nomogram could augment net benefits and demonstrate a wider range of threshold probabilities than the FAST score in the prediction of hemorrhagic stroke (Fig. 5).

## Discussion

Stroke is a major cause of death and long-term cognitive impairment in China [20]. Timely treatment is critical to the prognosis of stroke patients, which reduces mortality and improves neurological prognosis [21]. Efficient pre-hospital assessment is essential for EMS to differentiate between hemorrhagic and ischemic stroke [22]. Traditionally, ischemic and hemorrhagic stroke had the common risk factors. However, the risk factors for identifying the different subtypes of stroke are unclear.

This study established a practical and convenient tool based on the FAST score and combined with age, systolic blood pressure, hypertension, and vomiting to predict the risk of hemorrhagic stroke in patients with suspected stroke symptoms for EMS staff. All independent predictors were acquired in clinical practice easily and inexpensively out-of-hospital. This nomogram has proven clinical utility and is useful for risk decision-making in patients with hemorrhagic stroke during pre-hospital first aid.

We found that age was an important independent factor to distinguish hemorrhagic stroke from ischemic stroke. The incidence of stroke among young adults has increased in the past two decades [23]. The patients with hemorrhagic stroke were younger than ischemic stroke [24, 25]. One study of 1,880 non-fatal stroke patients in Japan found that the mean age was 74.1 years for ischemic stroke, and 68.2 years for hemorrhagic stroke [26]. Further, a recent study also confirmed that the median age of patients was 74 (66–82) years for ischemic stroke, 70 (59–79) years for intracerebral hemorrhage, and 64 (53–75) years for subarachnoid hemorrhage among the 183,080 stroke patients [27]. Thus, younger patients who suspected stroke may have an increased risk of hemorrhagic stroke prehospital, which was associated with the poorer blood pressure control and an increased proportion of subarachnoid hemorrhage [28].

At present, hypertension has been recognized as the most important risk factor affecting the occurrence of stroke [29, 30]. This may be related to cerebral vascular remodeling caused by the decrease in the diameter of the cerebrovascular lumen and the increase in the thickness of the vascular wall when hypertension occurs [31]. The elevated blood pressure that occurred in the hyperacute phase of stroke was often associated with sympathetic overactivity [32, 33]. Rawshani et al.’s study found that systolic blood pressure was a risk factor that affected cerebrovascular accidents [34]. Importantly, Katsanos confirmed that the lower the systolic blood pressure, the lower risk of hemorrhagic stroke happened, which was consistent with our results [35]. Therefore, we should pay more attention to uncontrolled systolic blood pressure in hypertensive patients, which induced the increased risk of hemorrhagic stroke [36]. Furthermore, we should focus on individual blood pressure treatment goals to reduce the risk of hemorrhagic stroke in hypertensive patients.

The FAST score is a traditional tool for identifying strokes with large vessel occlusion [37]. For patients suspected of acute stroke, questions including facial drooping, arm weakness, and slurred speech should be evaluated according to the FAST score [38, 39]. It was worth noting that stroke was the leading cause of adult-acquired disability [40]. In our study, we found that a higher incidence of slurred speech and arm weakness was observed in hemorrhagic stroke patients. Slurred speech was a manifestation of progressive central nervous system damage [19], always manifested as dysarthria, and was caused by weak, slow, or uncoordinated muscle control [41–43]. The appearance of arm weakness might be related to the regulation of hand function by the corticoreticulospinal tract [44]. Therefore, slurred speech and arm weakness played the important roles in the diagnosis of stroke, especially in hemorrhagic stroke.

Intracranial pressure could be increased after ischemic or hemorrhagic stroke [45, 46]. The typical clinical manifestations of elevated intracranial pressure were headache, vomiting, and even loss of consciousness [47]. Especially in hemorrhagic stroke, blood could extravasate into surrounding brain tissue due to blood vessel ruptures [48]. Our study also demonstrated that vomiting was an important clinical manifestation to distinguish hemorrhagic stroke. More importantly, in contrast to other symptoms, vomiting was a typical symptom that can be assessed even in patients with unconsciousness [49]. In addition, vomiting was the most common manifestation in children with hemorrhagic stroke [50], and it was rarely presented in children with ischemic stroke [51].

The AUC with nomogram was 0.790 in the training set and 0.808 in the validation set, which has moderate prediction efficiency. In addition, the AUC of the nomogram was superior to that of the FAST score in both sets. DCA curves showed that the nomogram demonstrated a wider range of threshold probabilities than the FAST score in the prediction of hemorrhagic stroke. Given the discriminative ability of the model, it was useful to identify the type of stroke early and optimize the nursing procedure.

Our study has several limitations. First, it was a retrospective single-center study, in which potential selection bias and recall bias were inevitable. Second, this study was not external validation because of the smaller sample size, and multicenter studies should be conducted future in verifying the clinical usefulness of the model. Furthermore, other stroke-related risk factors, such as alcohol consumption, smoking, and exercise habits were not included in our research, which were important factors affecting stroke and may have influenced our results.

## Conclusions

In summary, we identified several associated risk factors that could differentiate hemorrhagic and ischemic stroke prehospital. In addition, we developed a clinical nomogram based on the FAST score for differentiating hemorrhagic and ischemic stroke.

## Data Availability

The data and R codes are available from the corresponding author on reasonable request.
